# Novel *Frem1*-Related Mouse Phenotypes and Evidence of Genetic Interactions with *Gata4* and *Slit3*

**DOI:** 10.1371/journal.pone.0058830

**Published:** 2013-03-11

**Authors:** Tyler F. Beck, Oleg A. Shchelochkov, Zhiyin Yu, Bum Jun Kim, Andrés Hernández-García, Hitisha P. Zaveri, Colin Bishop, Paul A. Overbeek, David W. Stockton, Monica J. Justice, Daryl A. Scott

**Affiliations:** 1 Departments of Molecular and Human Genetics, Baylor College of Medicine, Houston, Texas, United States of America; 2 Molecular Physiology and Biophysics, Baylor College of Medicine, Houston, Texas, United States of America; 3 Molecular and Cell Biology, Baylor College of Medicine, Houston, Texas, United States of America; 4 Department of Pediatrics, The University of Iowa, Iowa City, Iowa, United States of America; 5 The Wake Forest Institute for Regenerative Medicine, Winston Salem, North Carolina, United States of America; 6 Departments of Pediatrics and Internal Medicine, Wayne State University, Detroit, Michigan, United States of America; National Cancer Institute, United States of America

## Abstract

The FRAS1-related extracellular matrix 1 (*FREM1*) gene encodes an extracellular matrix protein that plays a critical role in the development of multiple organ systems. In humans, recessive mutations in *FREM1* cause eye defects, congenital diaphragmatic hernia, renal anomalies and anorectal malformations including anteriorly placed anus. A similar constellation of findings–microphthalmia, cryptophthalmos, congenital diaphragmatic hernia, renal agenesis and rectal prolapse–have been described in FREM1-deficient mice. In this paper, we identify a homozygous *Frem1* missense mutation (c.1687A>T, p.Ile563Phe) in an N-ethyl-N-nitrosourea (ENU)-derived mouse strain, *crf11*, with microphthalmia, cryptophthalmos, renal agenesis and rectal prolapse. This mutation affects a highly conserved residue in FREM1’s third CSPG domain. The p.Ile563Phe change is predicted to be deleterious and to cause decreased FREM1 protein stability. The *crf11* allele also fails to complement the previously described *eyes2* allele of *Frem1* (p.Lys826*) providing further evidence that the *crf11* phenotype is due to changes affecting *Frem1* function. We then use mice bearing the *crf11* and *eyes2* alleles to identify lung lobulation defects and decreased anogenital distance in males as novel phenotypes associated with FREM1 deficiency in mice. Due to phenotypic overlaps between FREM1*-*deficient mice and mice that are deficient for the retinoic acid-responsive transcription factor GATA4 and the extracellular matrix protein SLIT3, we also perform experiments to look for *in vivo* genetic interactions between the genes that encode these proteins. These experiments reveal that *Frem1* interacts genetically with *Gata4* in the development of lung lobulation defects and with *Slit3* in the development of renal agenesis. These results demonstrate that FREM1-deficient mice faithfully recapitulate many of the phenotypes seen in individuals with FREM1 deficiency and that variations in GATA4 and SLIT3 expression modulate some FREM1-related phenotypes in mice.

## Introduction

The FRAS1-related extracellular matrix 1 (*FREM1*) gene encodes an extracellular matrix protein that plays a critical role in the development of multiple organs [1]. During epidermal development, FREM1 is secreted by mesenchymal cells into the basement membrane [2]. In the basement membrane, FREM1 forms a ternary complex with FRAS1 and FREM2, transmembrane proteins that are transported to the plasma membrane of epidermal cells with the help of GRIP1 and are shed from the cell surface by proteolytic processing [2]. In mice, recessive mutations affecting *Frem1* or *Frem2* lead to diminished expression of FREM1, FRAS1 and FREM2 in the basement membrane, suggesting that these proteins undergo reciprocal stabilization in this location [2]. Loss of the FREM1/FRAS1/FREM2 complex, due to recessive mutations in *Frem1*, *Fras1*, *Frem2* or *Grip1*, leads to a loss of epidermal integrity and the development of large fluid-filled blisters over the eyes and/or digits between E10.5 and E12.5. As a result, these mouse strains are often referred to collectively as ‘bleb’ mutants [3]–[9].

Given the known interactions between these proteins, it is not surprising that FREM1-, FRAS1-, FREM2- and GRIP1-deficient mice have overlapping patterns of defects that include cryptophthalmos and syndactyly–which are likely to be secondary effects of blister formation–and renal agenesis. Other features have only been documented in a subset of these mice. For example, abnormal lung lobulation has been documented in mice with mutations in *Fras1* and *Frem2* but have not been documented in mice with mutations in *Frem1* or *Grip1* [10], [11]. Similarly, recessive mutations in *Frem1* have been shown to cause congenital diaphragmatic hernia (CDH) which has not been documented in mice with *Fras1*, *Frem2* or *Grip1* mutations [12].

Similarities and differences are also seen in the human phenotypes associated with these genes. Recessive mutations in *FRAS1, FREM2,* and *GRIP1* cause Fraser syndrome which is characterized by cognitive impairments, cryptophthalmos, syndactyly, genital and renal anomalies and a range of other structural defects including CDH, lung lobulation defects and anal anomalies (OMIM #219000) [8], [13]–[18]. Recessive mutations in *FREM1* have not been implicated in the development of Fraser syndrome but have been found to cause two rare genetic syndromes, Bifid Nose with or without Anorectal and Renal anomalies syndrome (BNAR; OMIM #608980) and Manitoba OculoTrichoAnal syndrome (MOTA; OMIM #248450), that have significant clinical overlap with Fraser syndrome [19]–[25]. The spectrum of defects seen in BNAR and MOTA syndromes includes bifid or broad nasal tips, eye anomalies–cryptophthalmos, microphthalmia, anophthalmia and colobomas–aberrant hairlines extending towards the eye, omphalocele, renal agenesis, and anorectal malformations–anteriorly placed anus, anal stenosis, rectal atresia, and imperforate anus [19]–[25]. Although CDH has not been described in individuals with these syndromes, we have recently described an infant with isolated CDH who carries recessive mutations in *FREM1* [12].

In this report, we identify a homozygous *Frem1* missense mutation–c.1687A>T, p.Ile563Phe–in the N-ethyl-N-nitrosourea (ENU)-derived mouse strain *craniofacial 11* (*crf11*). The compound heterozygous progeny of crosses between *crf11* mice and *Frem1* ^eyes2/eyes2^ mice–which are homozygous for a truncating mutation in *Frem1* (p.Lys826*)–exhibit eye defects, CDH and renal agenesis indicating failure of complementation. We also find that *Frem1* ^crf11/crf11^, *Frem1* ^crf11/eyes2^ and *Frem1* ^eyes2/eyes2^ mice have lung lobulation defects and that male *Frem1* ^eyes2/eyes2^ mice on a C57BL6 background have decreased anogenital distances compared to wild-type littermate controls.

Like FREM1-deficient mice, mice that are deficient for the retinoic acid responsive transcription factor GATA4 have anterior CDH and lung lobulation defects [26]–[28]. Phenotypic similarities–CDH and renal agenesis–also exist between FREM1-deficient mice and mice with recessive mutations in *Slit3,* which encodes an extracellular matrix protein [29], [30]. This prompted us to look for *in vivo* genetic interactions between these genes. We found that *Frem1* and *Gata4* interact genetically in the development of lung lobulation defects and that *Frem1* and *Slit3* interact genetically in the development of renal agenesis.

## Materials and Methods

### Mouse Studies

All experiments using mouse models were conducted in accordance with the recommendations in the *Guide for the Care and Use of Laboratory Animals* of the National Institutes of Health. The associated protocols were approved by the Institutional Animal Care and Use Committee of Baylor College of Medicine (Animal Welfare Assurance #A3832-01).

All efforts were made to minimize suffering. Euthanasia was carried out using methods consistent with the recommendations of the Panel of Euthanasia of the American Veterinary Medical Association and included carbon dioxide (CO_2_) inhalation or an overdose of an inhaled anesthetic, such as isoflurane, in an appropriate enclosure.

### Generation of *crf11* Mice by N-ethyl-N-nitrosourea (ENU) Mutagenesis

ENU mutagenesis was carried out using 8- to 12-week-old male C57BL/6J mice given 300 mg/kg of N-ethyl-N-nitrosourea. ENU was administered in three 100 mg/kg intraperitoneal injections at 1-week intervals, as previously described [31]. These mice were then bred and intercrossed to screen for viable recessive phenotypes. The *craniofacial 11* (*crf11)* strain (MGI: 2671571) was identified based on the presence of unilateral and bilateral microphthalmia and/or cryptophthalmos and variable craniofacial defects [32].

### Mapping and Cloning of the *crf11* Allele

Mice from the *crf11* strain were backcrossed to 129S6/SvEvTac mice. The progeny of these crosses were intercrossed and mice carrying the *crf11* allele were identified based on their eye phenotypes. After several generations of backcrossing, *crf11* mice were genotyped using single nucleotide polymorphism (SNP) markers that discriminate between C57BL/6J and 129S6/SvEvTac strains. Linkage analysis was performed as previously described and the *crf11* allele was found to be linked to markers on mouse chromosome 4 [33]. Additional backcrosses were carried out and *crf11* mice were genotyped by amplifying and sequencing regions of the genome which harbored SNPs known to vary between the C57BL/6J and 129S6/SvEvTac strains.

Candidate genes from the *crf11* interval–including *Frem1*–were selected for further mutation screening. The coding region and associated intron/exon junctions of *Frem1* were amplified by PCR and the resulting amplification products were sequenced. Primer sequences are available on request. Sequence traces were analyzed using Sequencher 4.7 software (Gene Codes Corporation).

### Phenotypic Analysis of Mice Bearing the *crf11* and *eyes2* Alleles

Necropsies were performed on *Frem1* ^crf11/crf11^, *Frem1* ^crf11/eyes2^, and *Frem1* ^eyes2/eyes2^ mice to determine the spectrum and penetrance of *Frem1-*associated phenotypes on a mixed B6J/129S6 background. Similar studies were performed on a congenic *Frem1* ^eyes2/eyes2^ mouse line that had been backcrossed to C57BL6/J mice for at least 8 generations [12].

### *In situ* Hybridization and Immunohistochemistry

*In situ* hybridization probes were created to target *Frem1, Fras1* and *Frem2* transcripts. The probe targeting *Frem1* was designed using Sequencher 4.7 software (Gene Codes Corporation, Ann Arbor, MI, USA), while the probes targeting *Fras1* and *Frem2* were based on *in situ* probe designs from the Eurexpress database (http://www.eurexpress.org/ee/; *Frem2* template ID T38972; *Fras1* template ID T36392), as previously described [12]. *In situ* studies were performed by the IDDRC RNA *In Situ* Hybridization core at Baylor College of Medicine, on sagittal sections of E14.5, C57BL6/J embryos as previously described [12].

Immunohistochemistry was performed on paraffin sections of E14.5 wild-type C57BL6/J embryos using a mouse polyclonal primary antibody targeted against GATA4 (Sc-25310, Santa Cruz Biotechnology, Santa Cruz, CA) as previously described [27].

### Measurement of Fecal Diameter and Anogenital Distance

To minimize variation, measurements of fecal diameter and anogenital distance were measured on P28-P30 littermates on a C57BL/6J background by a single individual. Feces was collected from mice of different genotypes and imaged using a Nikon SM21500 microscope (Nikon Instruments, Melville, NY, USA) with an attached Zeiss Axiocam MRc camera (Carl Zeiss AG, Oberkochen, Germany). Fecal diameter was then measured using the AxioVision Release 4.6.1.0 software (Carl Zeiss AG). For measurements of anogenital distance, the anogenital regions of euthanized mice were pressed against a clear plastic surface and measured from the other side. In female mice, the anogenital distance was measured from the center of the anal opening to the center of the vaginal opening. In male mice, the anogenital distance was measured from the center of the anus to the base of the genital papilla.

### Statistics

Chi-square analyses were performed using the Opus12 chi-square calculator (http://www.opus12.org/Chi-Square_Calculator.html) to assess genotype- and strain-dependent differences in the penetrance of individual phenotypes. A Fisher Exact test was used for comparisons in which expected frequencies were less than five. For anogenital distance and fecal diameter studies, results were analyzed using one-way analysis of variance performed using the IBM SPSS Statistics software (IBM, Armonk, NY, USA).

## Results

### Homozygous *Frem1* Missense Mutation in *crf11* Mice

**Figure 1:**
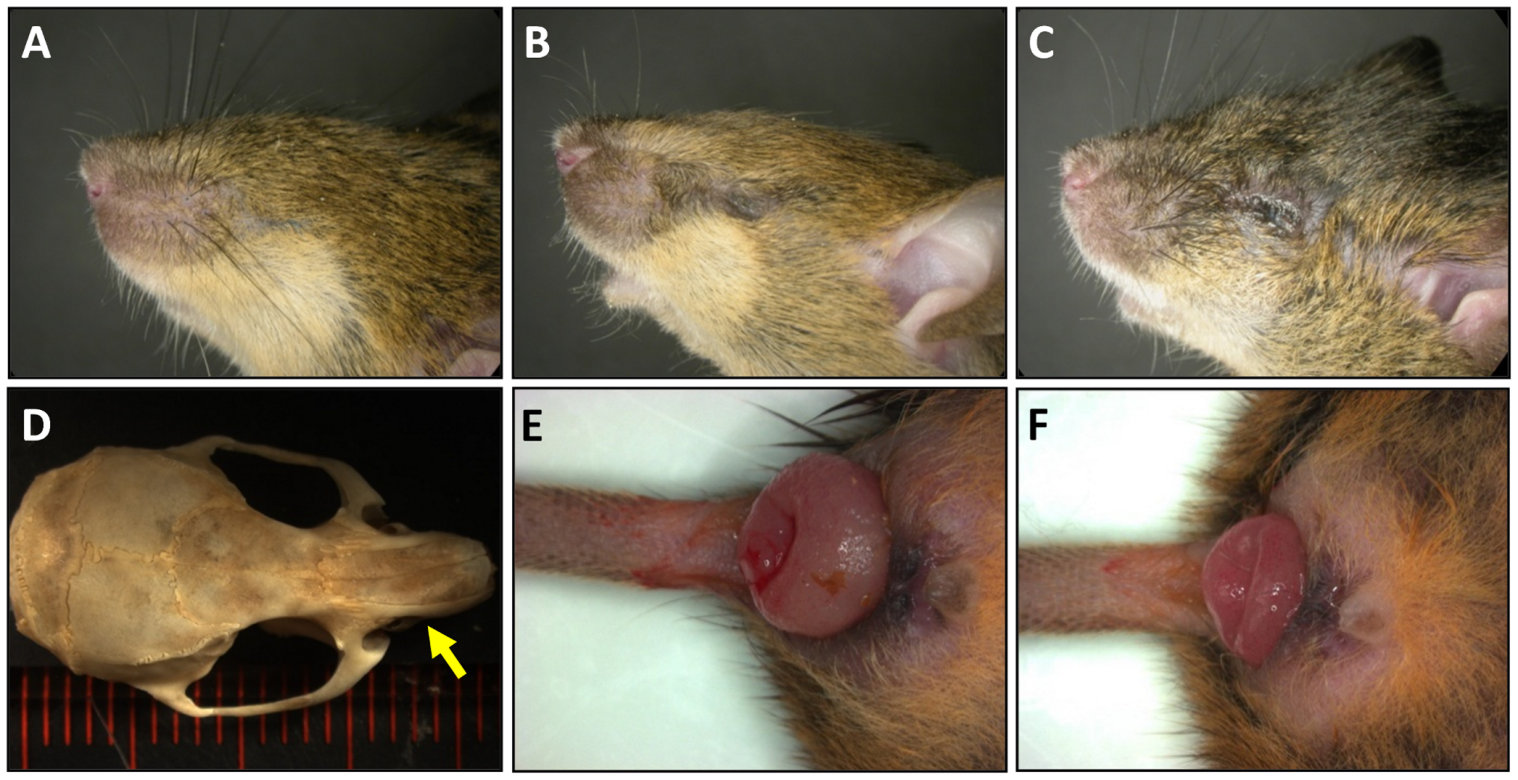
The phenotype of *crf11* mice includes eye defects, variable craniofacial defects, renal agenesis and rectal prolapse. A–C) *crf11* mice have a spectrum of eye defects that includes cryptophthalmos and microphthalmia. D) Deviation of the nasal bones (yellow arrow) is an example of the variable craniofacial defects seen in *crf11* mice. E–F) A subset of *crf11* mice exhibits renal agenesis (not shown) and *crf11* mice have a propensity to develop rectal prolapse as adults.

In a recessive ENU mutagenesis screen, we identified a mouse strain with a high penetrance of unilateral and bilateral cryptophthalmos and/or microphthalmia (18/18, 100%) and variable craniofacial defects (Fig. 1). The strain was named *craniofacial 11* (*crf11*; MGI: 2671571) [32]. Additional phenotyping of this strain revealed unilateral renal agenesis in 6.9% (4/58) and a propensity to develop rectal prolapse in adulthood (Fig. 1).

After several generations of backcrossing to 129S6/SvEvTac mice, the *crf11* phenotype was linked to a region of mouse chromosome 4. This region contained *Frem1* which was considered a promising candidate gene based on published reports of other FREM1-deficient mice with similar phenotypes (Table 1) [1], [2], [4], [7], [12].

**Table 1:** Phenotypes of Frem1-deficient mouse strains.

Strain designation	Mutation	Associated phenotypes	Reference
*Frem1* ^heb^	LINE-1 insertion into exon 17	Eye anomalies, syndactyly	Varnum et al. 1981 [7]; Smyth et al. 2004 [1]
*Frem1^bat^*	Splice site mutation, exon 25	Eye anomalies, craniofacial abnormalities, renal agenesis, rectal prolapse, syndactyly	Smyth et al. 2004 [1]; Vissers et al. 2011 [63]; Slavotinek et al. 2011 [21]
*Frem1* ^bfd^	Mutation not identified	Eye anomalies, craniofacial abnormalities, renal agenesis, syndactyly	Smyth et al. 2004 [1]
*Frem1* ^tm1Ksek^	Targeted replacement of exon 2 with a Neo cassette	Eye anomalies, craniofacial abnormalities, renal agenesis, syndactyly	Kiyozumi et al. 2006 [2]; Vissers et al. 2011 [63]; Kiyozumi et al. 2012 [64]
*Frem1* ^eyes2^	c.2477T>A, p.Lys826*	Eye anomalies, congenital diaphragmatic hernia,lung lobulation defects, renal agenesis, rectalprolapse, decreased anogenital distance in males	Kile et al. 2003 [32]; Beck et al. 2012 [12]; Current report
*Frem1* ^crf11^	c.1687A>T, p.Ile563Phe	Eye anomalies, craniofacial abnormalities, lung lobulation defects, renal agenesis, rectal prolapse	Kile et al. 2003 [32]; Current report

**Figure 2:**
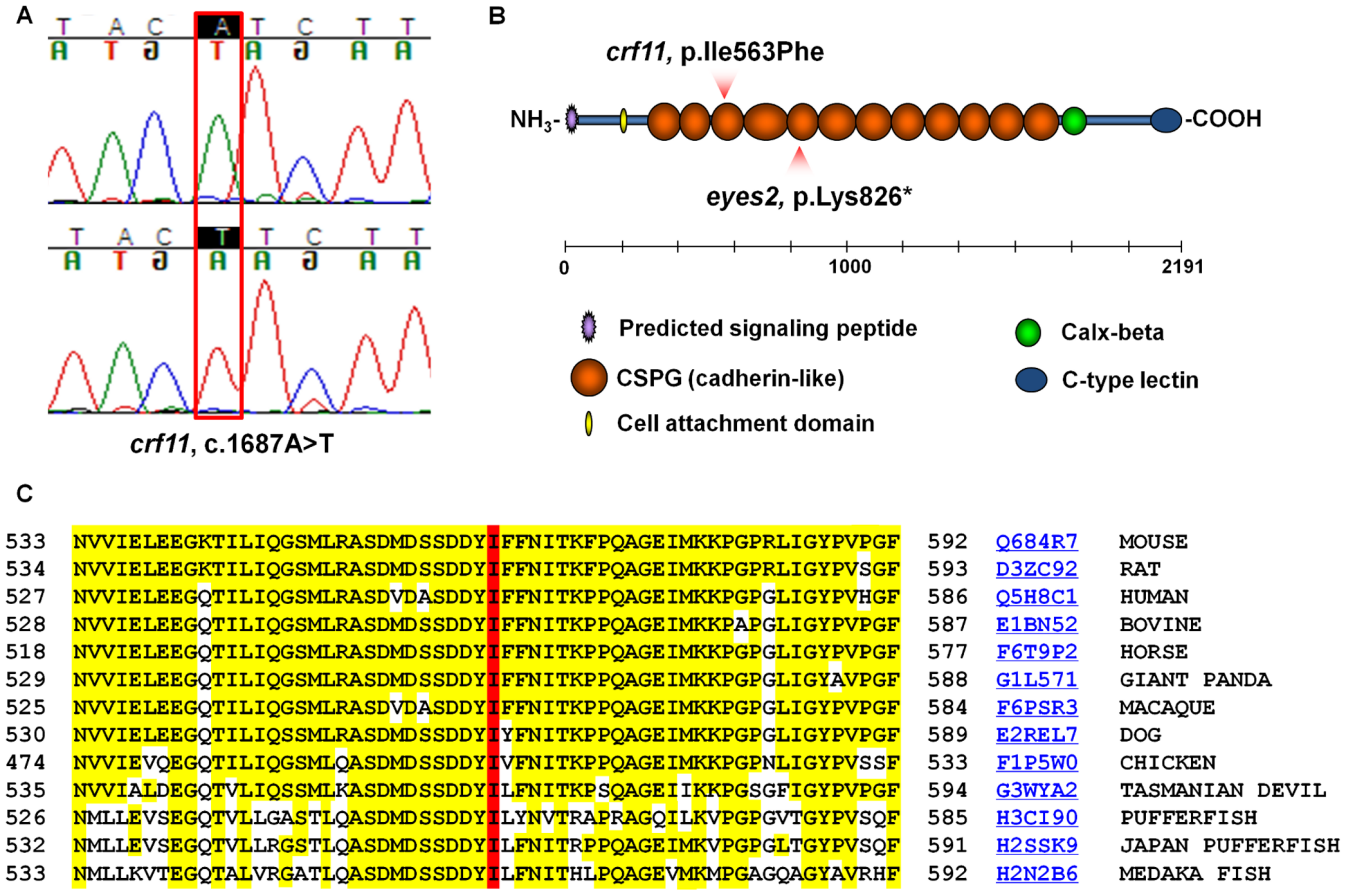
The *crf11* phenotype is due to a homozygous missense mutation in *Frem1* that affects a highly conserved amino acid in FREM1’s third CSPG domain. A) Sequencing of the *Frem1* gene in DNA from *crf11* mice revealed a homozygous c.1687A>T change that was not seen in control DNA. B) This change causes a single amino acid change (p.Ile563Phe) in the third CSPG domain of FREM1. This mutation is predicted to be damaging and to cause a large decrease in protein stability. The location of this change, and the *eyes2* nonsense mutation (p.Lys826*), are depicted in relation to the domains of the FREM1 protein. C) The isoleucine at position 563 in the mouse FREM1 protein is highly conserved through multiple species (highlighted in red). The conserved amino acids around this position are highlighted in yellow. The UniProt ID numbers of protein sequence used in this alignment are written in blue.

Sequencing of the *Frem1* coding region and intron/exon boundaries revealed a homozygous c.1687A>T change in DNA samples from *crf11* mice which was not found in DNA from C57BL/6J and 129S6/SvEvTac control mice (Fig. 2A). This change causes an isoleucine to phenylalanine change (p.Ile563Phe) at a highly conserved amino acid residue in FREM1’s third CSPG domain (Figs. 2B & 2C). CSPG domains are cadherin repeat-like repetitive units which are a hallmark of the FRAS-FREM protein family [1]. The p.Ile563Phe change was predicted to be “Non Tolerated” by SIFT (http://sift.jcvi.org/) and damaging by PANTHER PSEC (http://www.pantherdb.org/tools/csnpScoreForm.jsp), two online programs that predict the effects of amino acid substitutions on protein function [34], [35].

Since decreased stability is a major consequence of many pathogenic missense mutations, we used a variety of online tools to determine if the p.Ile563Phe change in FREM1 could lead to decreased protein stability [36]–[38]. Using the Scpred program (http://www.enzim.hu/scpred/scpred.html), we found that the isoleucine at amino acid position 563 lies within a predicted protein stabilization center [39]. The p.Ile563Phe mutation was subsequently predicted to cause a free energy change of –1.62 Kcal/mol by the I-Mutant ΔΔG program (http://gpcr2.biocomp.unibo.it/cgi/predictors/I-Mutant3.0/I-Mutant3.0.cgi; Temp 37°C; pH 7), consistent with a large decrease in protein stability [37], [40]. The MUPro program (http://mupro.proteomics.ics.uci.edu/) also predicted that this mutation leads to decreased protein stability [41].

### The *crf11* Allele Fails to Complement the *eyes2* Allele of *Frem1*

To confirm that the phenotype of the *crf11* strain was due to changes affecting *Frem1* function, we crossed *crf11* mice with *eyes2* mice which are homozygous for a previously described nonsense mutation in *Frem1* (p.Lys826*) [12]. The phenotypes seen in the resulting compound heterozygous progeny (*Frem1* ^crf11/eyes2^) are summarized in Table 2 and include unilateral and bilateral cryptophthalmos and/or microphthalmia, CDH and renal agenesis.

**Table 2:** The *crf11* and *eyes2* alleles of *Frem1* fail to complement.

Phenotypes	Prevalence in *Frem1* ^crf11/eyes2^ mice
Cryptophthalmos/microphthalmia	21/63 (33.3%)
Lung lobulation defects	22/63 (34.9%)
Congenital diaphragmatic hernia	1/63 (1.6%)
Renal agenesis	2/63 (3.2%)

This failure of the *crf11* allele to complement the *eyes2* allele of *Frem1* suggests that an ENU-induced defect in the *Frem1* gene is the cause of the *crf11* phenotype. Since the *crf11* and *eyes2* mouse lines were created in the same mutagenesis screen, and have been maintained on similar B6J/129S6 backgrounds, it is less likely that failure to complement is due, instead, to strain specific variants linked to the mutant locus [12], [32], [42]. However, these studies cannot rule out the possibility that the c.1687A>T change is not the cause of the *crf11* phenotype but is, instead, linked to a causative mutation which was not identified in our sequencing analysis.

### *Frem1* Mutant Mice Exhibit Lung Lobulation Defects

**Figure 3:**
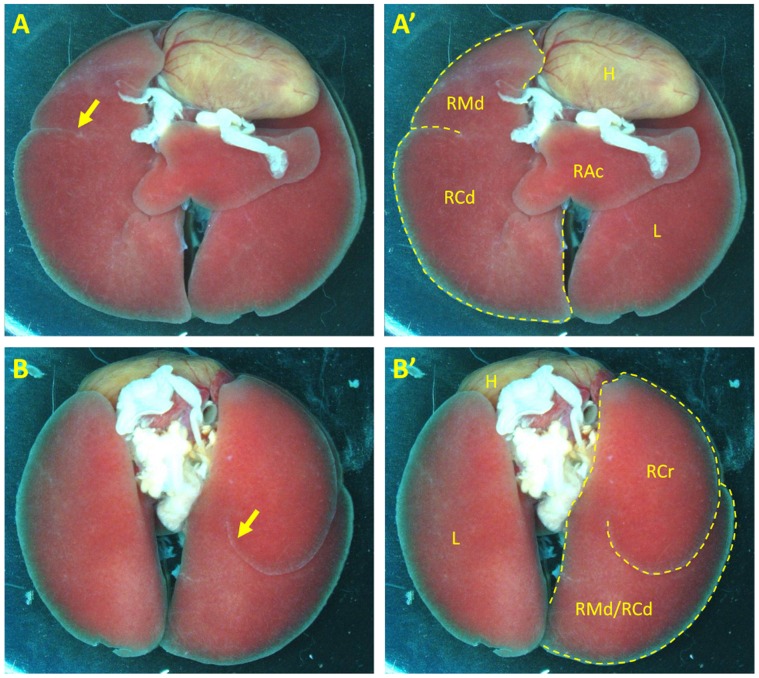
FREM1 deficiency causes lung lobulation defects in mice. (A and A′) Ventral view of the lungs of a *Frem1* ^eyes2/eyes2^ mouse in which the right middle (RMd) and right caudal lobe (RCd) are fused with only small, partial fissures defining the boundaries of each lobe (yellow arrow). (B and B′) Dorsal view of the same lungs showing partial fusion of the right cranial lobe (RCr) to the fused right middle and caudal lobes (RMd/RCd) with an incomplete fissure separating these lobes (yellow arrow). In A′ and B′, the right lung lobes are outlined with a dashed yellow line. The left lung (L) and the right accessory lobe (RAc) are normal. H = heart.

In addition to the phenotypes previously described in FREM1-deficient mice, we noted that lung lobulation defects were common in *Frem1* ^crf11/crf11^ (13/58, 22.4%), *Frem1* ^crf11/eyes2^ (22/63, 34.9%) and *Frem1* ^eyes2/eyes2^ (11/39, 28.2%) mice on a mixed B6Brd/129S6 background. These defects consisted of fusions between the cranial, medial and caudal lobes of the right lung. In severe cases, all of the lobes were partially or completely fused (Fig. 3). The right accessory lobe and the left lung appeared normal in all cases. The rate of lung lobulation defects was significantly higher in *Frem1* ^eyes2/eyes2^ mice on C57BL/6J background (55/73, 75.3%) compared to *Frem1* ^eyes2/eyes2^ mice on a mixed B6Brd/129S6 background (75.3% vs. 28.2%, p<0.0001). This suggests that genetic factors can modify the penetrance of this phenotype.

### Expression of *Frem1*, *Fras1*, *Frem2* in the Developing Lung

Lung lobulation defects have been reported previously in FRAS1-deficient and FREM2-deficient mice [10], [11]. In the case of FRAS1-deficient mice, branching morphogenesis–the process by which reciprocal interactions between the epithelium and its underlying mesenchyme produce the individual branches of the bronchial tree–was found to be normal [10], [43], [44]. Further evaluations performed on embryos at E14.5, suggested that defects in the basement membrane of the visceral pleura surrounding the developing lung were the underlying cause of the lung lobe fusions seen in FRAS1-deficient mice [10].

**Figure 4:**
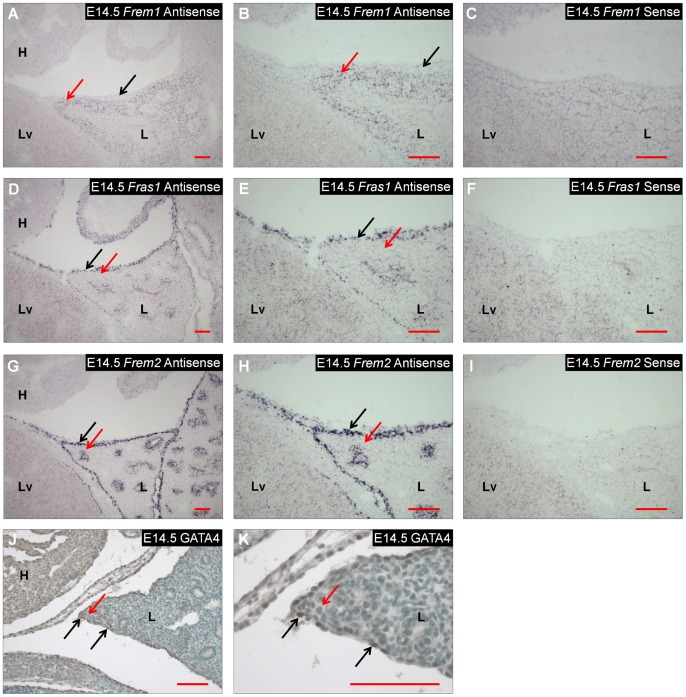
*Frem1, Fras1, Frem2* and GATA4 are expressed in the developing lung at E14.5. A–I) The expression of *Frem1, Fras1* and *Frem2* was determined in sagittal sections from wild-type E14.5 embryos using *in situ* hybridization. In each case, a sense probe was used as a negative control. A–C) Expression of *Frem1* is seen in the mesenchymal cells of the developing lung (red arrows) but is not detected in the mesothelial cells of the parietal pleura (black arrows). D–I) *Fras1* (panels D–F) and *Frem2* (panels G–I) are not expressed in the mesenchymal cells of the developing lung (red arrows) but are expressed in the upper, mesothelial cells of the parietal pleura (black arrows). J–K) GATA4 immunostaining on sagittal sections from wild-type E14.5 embryos showed no evidence of GATA4 nuclear staining in mesenchymal cells of the developing lung (red arrow) but strong nuclear staining in the mesothelial cells of the parietal pleura (black arrows). Red scale bar = 100 µm. H = heart; L = lung; Lv = Liver.

If similar defects underlie the lung lobulation defects in FREM1 and FREM2-deficient mice, we would expect that *Frem1* and *Frem2* would be expressed along with *Fras1* in cells adjacent to the basement membrane of the visceral pleura. To determine the expression patterns of these genes in the developing lung, we performed *in situ* hybridization studies on wild-type embryos at E14.5. At this time point, *Frem1* transcripts were detected in the underlying mesenchymal cells of the developing lung but not in the mesothelial cells of visceral pleura (Fig. 4A–C). In contrast, *Fras1* and *Frem2* transcripts were detected in the mesothelial cells of visceral pleura but were not detected in the underlying mesenchymal cells (Fig. 4D–I). This pattern is identical to the expression pattern of these genes in the developing diaphragm [12]. A similar expression pattern is also seen in the developing skin, where FREM1 is secreted by mesenchymal cells into the basement membrane and FRAS1 and FREM2 are released into the basement membrane from epidermal cells [2].

### Male FREM1-deficient Mice have Decreased Anogenital Distances

**Figure 5:**
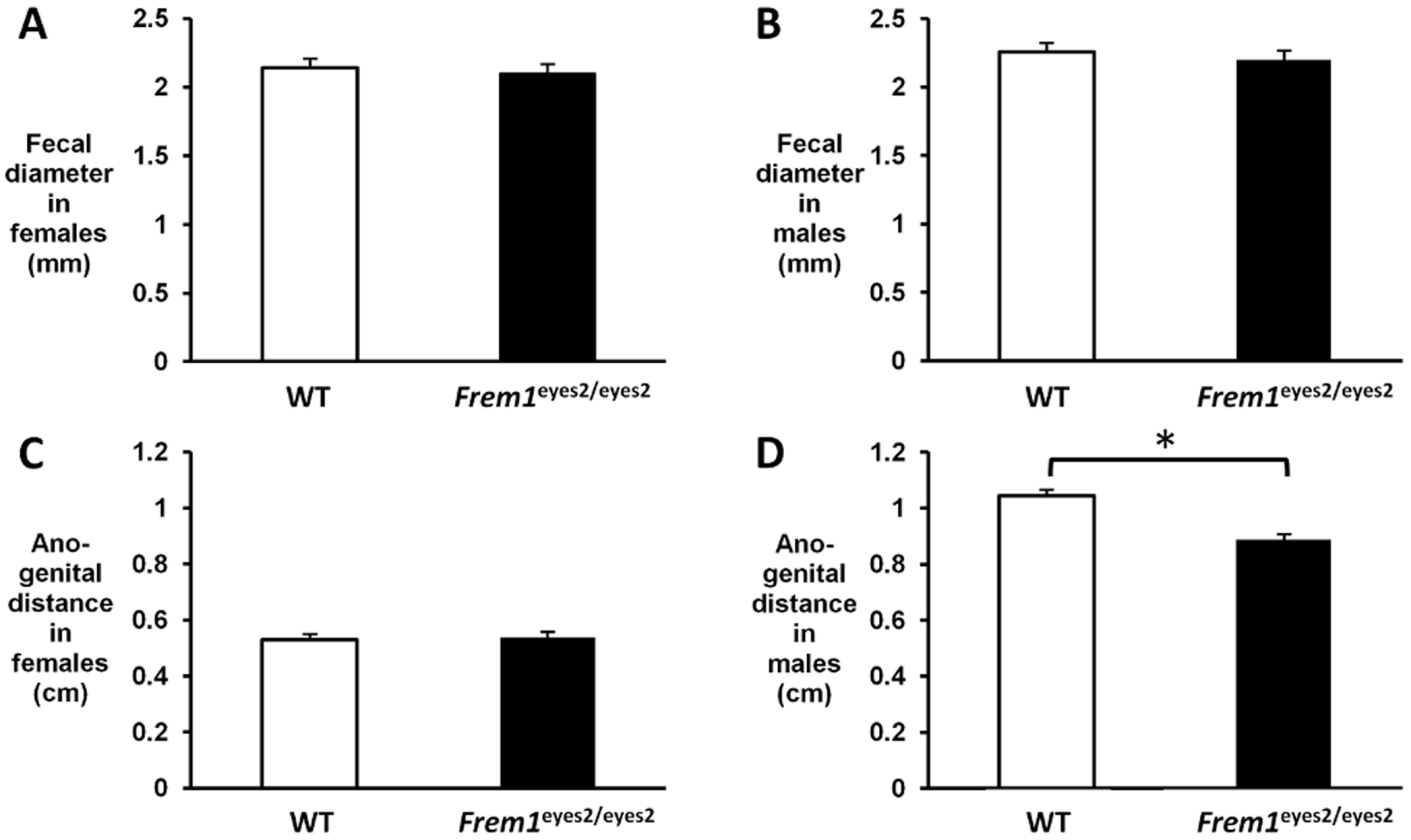
Male *Frem1* ^eyes2/eyes2^ mice on a C57BL6 background have decreased anogenital distance. Comparisons of fecal diameter and anogenital distance were made between female and male P28–P30 *Frem1* ^eyes2/eyes2^ mice and their sex-matched wild-type littermates on a C57BL6 background. A–B) No significant difference was seen in the fecal diameters of female or male *Frem1* ^eyes2/eyes2^ mice compared to their wild-type littermates. C) No significant difference was seen in the anogenital distances of female *Frem1* ^eyes2/eyes2^ mice compared to their wild-type littermates. D) The anogenital distances of male *Frem1* ^eyes2/eyes2^ mice was significantly smaller than that of their wild-type littermates (p = 0.026) despite having similar weights and lengths. Error bars show standard error of the mean.

Individuals with BNAR syndrome have a spectrum of anorectal malformations that includes anal stenosis and anteriorly placed anus [19]. To determine if the development of rectal prolapse in FREM1-deficient mice was due, in part, to anal stenosis or anteriorly placed anus, we compared the fecal diameters and anogenital distances of P28–P30 *Frem1* ^eyes2/eyes2^ mice and their wild-type littermates on a C57BL/6J background. No significant differences in fecal diameter were found between mice of these genotypes (Fig. 5A & 5B). While no difference was seen in the anogenital distance of female *Frem1* ^eyes2/eyes2^ mice and their female wild-type littermates (Fig. 5C), a significant difference in average anogenital distance was found between male *Frem1* ^eyes2/eyes2^ mice and their male wild-type littermates (0.89 cm vs. 1.05 cm, p = 0.026) (Fig. 5D). This difference was not attributable to a difference in overall size since the average weight and length of P28–P30 wild-type and *Frem1* ^eyes2/eyes2^ mice on this background are not significantly different (14.4 g vs. 15.5 g, p = 0.58; 14.8 cm vs. 14.9 cm, p = 0.6).

### *Frem1* and *Gata4* Interact Genetically in the Development of Lung Lobulation Defects

*Gata4* ^+/Δex2^ mice, which harbor a deletion in exon 2 of *Gata4*, have anterior congenital diaphragmatic hernias that are similar to those seen in FREM1-deficient mice [12], [26]. Defects in *Gata4* also cause lung lobulation defects. Specifically, embryos that are homozygous for the V238G missense mutation in *Gata4* (*Gata4* ^ki/ki^), which die between E11.5 and E13.5, have abnormal lung lobulation with failure of accessory lobe development being evident in cultured lungs harvested at E11.5 and E12.5 [28]. Loss of the accessory lobe in *Gata4* ^ki/ki^ mice is attributable to defects in branching morphogenesis.

To determine if *Frem1* and *Gata4* interact genetically, we performed crosses between *Frem1* ^eyes2/eyes2^ and a *Gata4* ^+/−^ mouse strain described by Molkentin et al. to obtain *Frem1* ^eyes2/+^;*Gata4* ^+/−^ mice [45]. These double heterozygous mice were then crossed to *Frem1* ^eyes2/eyes2^ mice to generate progeny with four different genotypes (*Frem1* ^eyes2/+^, *Frem1* ^eyes2/+^;*Gata4* ^+/−^, *Frem1* ^eyes2/eyes2^, *Frem1* ^eyes2/eyes2^;*Gata4* ^+/−^) on a mixed B6/129S6 background. The number of mice of each genotype recovered at weaning (P28) and the prevalence of phenotypes associated with each genotype are summarized in Table 3.

**Table 3:** Effects of *Gata4* haploinsufficiency on the prevalence of *Frem1-*associated phenotypes.

	*Frem1* ^eyes2/+^	*Frem1* ^eyes2/+^;*Gata4* ^+/−^	*Frem1* ^eyes2/eyes2^	*Frem1* ^eyes2/eyes2^;*Gata4* ^+/−^
Recovery at weaning (% of Total)	77/214 (36.0%)	47/214 (22.0%)	61/214 (28.5%)	29/214 (13.6%)
Congenital diaphragmatic hernia	0/77 (0%)	0/47 (0%)	3/61 (4.9%)	0/29 (0%)
Lung lobe fusion	0/77 (0%)	2/47 (4.3%)	12/61 (19.7%)	16/29 (55.2%)
Renal agenesis	0/77 (0%)	0/47 (0%)	4/61 (6.6%)	2/29 (6.9%)

The expected frequency of each genotype in this cross was 25%. However, progeny were not recovered in Mendelian ratios at weaning (p<0.0001). Although the difference in the number of progeny with *Frem1* ^eyes2/+^ and *Frem1* ^eyes2/eyes2^ genotypes did not reach statistical significance (77/214 vs. 61/214, p = 0.173), a statistically significant decrease was seen between the number of progeny with a *Frem1* ^eyes2/+^;*Gata4* ^+/−^ genotype and the number of progeny with a *Frem1* ^eyes2/eyes2^;*Gata4* ^+/−^ genotype (47/214 vs. 29/214, p = 0.039). This was not surprising, since embryonic lethality has been previously documented in FREM1-deficient mice and is thought to occur as a result of blister formation with subsequent hemorrhage [1], [4], [7]. A statistically significant decrease in the number of progeny was seen with the addition of the *Gata4* null-allele to both the *Frem1* ^eyes2/+^ genotype (77/214 vs. 47/214, p<0.01) and the *Frem1* ^eyes2/eyes2^ genotype (47/214 vs. 29/214, p<0.001). This is consistent with the increased mortality which has previously been reported in *Gata4* ^+/−^ mice [45].

CDH and renal agenesis were not seen in *Frem1* ^eyes2/+^ or *Frem1* ^eyes2/+^;*Gata4* ^+/−^ mice, nor was an increase in the prevalence of these defects detected between *Frem1* ^eyes2/eyes2^ and *Frem1* ^eyes2/eyes2^;*Gata4* ^+/−^ mice. While no *Frem1* ^eyes2/+^ mice had abnormal lung lobulation, 4.3% (2/47) of *Frem1* ^eyes2/+^;*Gata4* ^+/−^ mice had evidence of fused lung lobes. A significant difference in the prevalence of lung lobulation defects was detected between *Frem1* ^eyes2/+^;*Gata4* ^+/−^ and *Frem1* ^eyes2/eyes2^;*Gata4* ^+/−^ mice (4.3% vs. 55.2%, p<<0.001) and between *Frem1* ^eyes2/eyes2^ and *Frem1* ^eyes2/eyes2^;*Gata4* ^+/−^ mice (19.7% vs. 55.2%, p<0.001). In all cases, the lung lobe fusions were similar to those seen in *Frem1* ^crf11/crf11^ and *Frem1* ^eyes2/eyes2^ mice and did not involve the right accessory lobe or the left lung. These results suggest that *Frem1* and *Gata4* interact genetically in the development of lung lobulation defects, since this increase in prevalence is more than can be attributed to the additive effects of homozygosity for the *eyes2* allele (19.7%) and the potential contribution of *Gata4* haploinsufficiency (4.3%) alone.

To determine if *Gata4* and *Frem1* are expressed in the same cells of the lung, we performed immunohistochemical analyses of GATA4 expression at E14.5. These studies revealed GATA4 nuclear staining in the mesothelial cells of the visceral pleura, where *Fras1* and *Frem2* are expressed (Figs. 4J & 4K), but little evidence of GATA4 nuclear staining in the mesenchymal cells of the lung where *Frem1* is expressed. This is consistent with previous published reports in which GATA4 expression was found mainly in the mesothelial cells of the developing lungs at E11.5, a time point after secondary lung branches have been established [28].

### *Frem1* and *Slit3* Interact Genetically in the Development of Renal Agenesis

Mice that lack the extracellular matrix protein SLIT3 develop a central form of CDH and have a high rate of renal agenesis [29], [30]. To determine if *Frem1* and *Slit3* interact genetically in the development of CDH, renal agenesis and/or lung lobulation defects, we crossed *Frem1* ^eyes2/eyes2^ mice to mice in which one copy of the *Slit3* gene has been disrupted by insertion of a *Sleeping Beauty*-tyrosinase transposon (*Slit3* ^+/−^) [46]. Mice that are homozygous for this *Slit3* allele have renal agenesis and CDH and usually die at birth or shortly thereafter. After obtaining *Frem1* ^eyes2/+^; *Slit3* ^+/−^ progeny, we crossed them to *Frem1* ^eyes2/eyes2^ mice to obtain progeny of four different genotypes (*Frem1* ^eyes2/+^, *Frem1* ^eyes2/+^;*Slit3* ^+/−^, *Frem1* ^eyes2/eyes2^, *Frem1* ^eyes2/eyes2^;*Slit3* ^+/−^) on a mixed FVB-N/C57BL6/129S6 background. The number of mice of each genotype recovered at weaning (P28) and the prevalence of phenotypes associated with each genotype are summarized in Table 4.

**Table 4:** Effects of *Slit3* haploinsufficiency on the prevalence of *Frem1-*associated phenotypes.

	*Frem1* ^eyes2/+^	*Frem1* ^eyes2/+^;*Slit3* ^+/−^	*Frem1* ^eyes2/eyes2^	*Frem1* ^eyes2/eyes2^;*Slit3* ^+/−^
Recovery at weaning (% of Total)	110/390 (28.2%)	126/390 (32.3%)	73/390 (18.7%)	81/390 (20.7%)
Congenital diaphragmatic hernia	0/110 (0%)	0/126 (0%)	2/73 (2.7%)	1/81 (1.2%)
Lung lobe fusion	1/110 (0.9%)	0/126 (0%)	16/73 (21.9%)	26/81 (32.1%)
Renal agenesis	0/110 (0%)	0/126 (0%)	2/73 (2.7%)	11/81 (13.6%)

The expected frequency of each genotype in this cross was 25%. However, progeny were not recovered in Mendelian ratios at weaning (p<0.001). A statistically significant decrease was seen between the number of progeny with a *Frem1* ^eyes2/+^ genotype and a *Frem1* ^eyes2/eyes2^ genotype (110/390 vs. 73/390, p<0.01) and between the number of progeny with a *Frem1* ^eyes2/+^;*Slit3* ^+/−^ genotype and a *Frem1* ^eyes2/eyes2^;*Slit3* ^+/−^ genotype (126/390 vs. 81/390, p<0.01). This is consistent with the increased mortality previously seen in FREM1-deficient mice [1], [4], [7]. In contrast, a statistically significant change in the number of progeny was not seen with the addition of the disrupted *Slit3* allele to either the *Frem1* ^eyes2/+^ genotype (110/390 vs. 126/290, p = 0.30) or the *Frem1* ^eyes2/eyes2^ genotype (73/390 vs. 81/390, p = 0.52).

CDH, renal agenesis and lung lobulation defects were not seen in any of the *Frem1* ^eyes2/+^ or *Frem1* ^eyes2/+^;*Slit3* ^+/−^ mice with the exception of one *Frem1* ^eyes2/+^ mouse in which lung lobulation defects were identified. Retrosternal CDH was detected at a low level in both *Frem1* ^eyes2/eyes2^ and *Frem1* ^eyes2/eyes2^;*Slit3* ^+/−^ mice with no statistically significant difference in CDH prevalence being seen between genotypes (2.7% vs. 1.2%, p = 0.60). The prevalence of lung lobulation defects increased between *Frem1* ^eyes2/eyes2^ and *Frem1* ^eyes2/eyes2^;*Slit3* ^+/−^ mice, but was not statistically significant (21.9% vs. 32.1%, p = 0.16).

Renal agenesis was not seen in *Frem1* ^eyes2/+^ mice (0/110) or in *Frem1* ^eyes2/+^;*Slit3* ^+/−^ mice (0/126). However, a statistically significant increase in the prevalence of renal agenesis was seen between *Frem1* ^eyes2/eyes2^ and *Frem1* ^eyes2/eyes2^;*Slit3* ^+/−^ mice (2.7% vs. 13.6%, p<0.02). These results suggest that *Frem1* and *Slit3* interact genetically in the development of renal agenesis, since this increase in prevalence is more than can be attributed to the additive effects of homozygosity for the *eyes2* allele (2.7%) and heterozygosity for the disrupted *Slit3* allele (0%) alone.

## Discussion

### Deleterious Mutations in *Frem1* cause the *crf11* Phenotype

The ENU-derived mouse line *crf11* displays several phenotypes which mimic previously described models of *Frem1* deficiency (Table 1). Sequencing of the *Frem1* gene revealed a homozygous missense mutation (c.1687A>T, p.Ile563Phe) that affects a highly conserved residue in FREM1’s third CSPG domain. The CSPG domains of chondroitin sulfate proteoglycan 4 (CSPG4) have been shown to interact with various growth factors and collagen molecules [47]–[50]. It is possible that the CSPG domains of FREM1 play a similar role.

Since the c.1687A>T, p.Ile563Phe mutation was predicted to be deleterious and to result in decreased protein stability, and the *crf11* allele failed to complement the *eyes2* allele of *Frem1* (p.Lys826*), we conclude that homozygosity for this change is the cause of the *crf11* phenotype [12], [32].

### Recessive Mutations in *Frem1* Cause Lung Lobulation Defects

Lung lobulation defects have not been described previously in FREM1-deficient mice. However, we found complete and partial fusions between the cranial, middle and caudal lobes of the right lung in *Frem1* ^crf11/crf11^, *Frem1* ^crf11/eyes2^ and *Frem1* ^eyes2/eyes2^ mice on a mixed B6Brd/129S6 background. This demonstrates that FREM1 plays a critical role in normal lung lobulation.

The lung lobulation defects seen in FREM1-deficient mice mirror those found in FRAS1 and FREM2-deficient mice. In FRAS1-deficient mice, defects in the basement membrane of the visceral pleura surrounding the developing lung have been implicated in the development of lung lobe fusions [10]. Since FREM1, FRAS1 and FREM2 form a ternary structure that is required for maintenance of epidermal integrity, it is possible that they also work together in the basement membrane of the visceral pleura to modulate lung lobe development. In support of this hypothesis, we have shown that *Frem1* is expressed in the mesenchymal cells that underlie the basement membrane of the visceral pleura and that *Fras1* and *Frem2* are expressed in the mesothelial cells of the visceral pleura at E14.5. We note that FREM1 and FRAS1 proteins have also been detected previously in the visceral pleura at E14.5 [51]. Taken together, these data suggest that FREM1 protein from mesenchymal cells and FRAS1 protein from mesothelial cells may be secreted into the basement membrane of the visceral pleura in a manner analogous to that documented in the mesenchymal and epidermal cells of the developing skin [2]. Although the presence of FREM2 protein in the basement membrane of the visceral pleura has not been demonstrated, the expression pattern of *Frem2* suggests that it may also be secreted from mesothelial cells into the basement membrane.

Lung lobulation defects have yet to be documented in individuals with FREM1 deficiency. However, in one large literature review, abnormal lung lobulation was documented in 5/117 (4.3%) of cases of Fraser syndrome, which can be caused by recessive mutations in *FRAS1, FREM2* and *GRIP1* [8], [13], [52]. Since lung lobulation defects are typically asymptomatic–and their diagnosis is often made incidentally–it is possible that such defects are also present in a subset of individuals with FREM1 deficiency but have gone undetected.

### FREM1 Deficiency Causes Reduced Anogenital Distance in Mice

Anal stenosis and anteriorly placed anus are among the anorectal anomalies seen in individuals with FREM1 deficiency [19]–[21]. While we found no evidence for anal stenosis in studies of fecal diameter, we show that male *Frem1* ^eyes2/eyes2^ mice on a C57BL6 background have decreased anogenital distances. Anteriorly placed anus is commonly associated with chronic constipation which may, in turn, predispose to rectal prolapse through excessive straining during defecation [53]. This suggests that reduced anogenital distance may contribute to the development of the rectal prolapse in FREM1-deficient mice. However, it is unlikely that abnormal anal position is the only factor that leads to a propensity to develop rectal prolapse in FREM1-deficient mice, since this propensity is seen in both males and females and no differences were seen in the anogenital distances of female *Frem1* ^eyes2/eyes2^ mice compared to their wild-type littermate controls.

Anogenital distance is a sexually dimorphic trait in rodents and humans with males having an anogenital distance that is 2 to 2.5 times larger than in females [54]. In rodents, male anogenital distance has been shown to be dependent on exposure and response to androgens during a “masculinization programming window” which has not been well defined in mice but is thought to occur between E13.5 and E17.5 based on observations in rats [55]–[57]. The dependence of male perineal development on the ability to produce and respond to androgens–particularly testosterone–may explain why male *eyes2* mice have reduced anogenital distances compared to their wild-type littermates while the anogenital distances of female *eyes2* and wild-type mice are indistinguishable.

A complete inability to respond to androgens–as seen in complete androgen insensitivity syndrome (OMIM #300068)–can cause male fetuses to develop female external genitalia [58]. Since *eyes2* male mice have male external genitalia and are fertile, we can assume that they are able to produce and respond to androgens *in utero*. However, further studies will be needed to determine if male *eyes2* embryos and mice have lower levels of androgen production or attenuated responses to androgens when compared to their sex-matched wild-type littermates.

Although decreased anogenital distance has not been documented in FRAS1, FREM2, or GRIP1-deficient mice, one large literature review showed that anteriorly placed or displaced anus was documented in 7/117 (6%) of individuals with Fraser syndrome, which can be caused by the genes that encode these proteins [15]. The spectrum of anorectal anomalies–which can also include anal stenosis, rectal atresia, and imperforate anus–is also similar between individuals with FREM1 deficiency and Fraser syndrome [15], [19]–[25]. This suggests that FREM1, FRAS1, FREM2 and GRIP1 may function together to ensure normal anorectal development.

### *Gata4* as a Modifier of Lung Lobulation Defects in FREM1-deficient Mice

We have previously reported a statistically significant difference between the prevalence of CDH in *Frem1* ^eyes2/eyes2^ mice on an inbred mixed B6Brd/129S6 background and on a C57BL/6J background [12]. In this report we have shown that similar differences exist in the prevalence of lung lobulation defects in *Frem1* ^eyes2/eyes2^ mice on these backgrounds. These findings provide evidence of the existence of genetic factors that influence the penetrance of CDH and lung lobulation defects caused by FREM1 deficiency [12].

Since GATA4-deficient mice have CDH and lung lobulation defects, we looked for evidence of a genetic interaction between *Frem1* and *Gata4* [12], [26]. Although no evidence of a genetic interaction was seen in the development of CDH, the prevalence of lung lobulation defects increased significantly in *FREM1* ^eyes2/eyes2^ mice with the addition of a *Gata4*-null allele, from 19.7% to 55.2% (p<0.001). This increase was more than could be attributed to the additive effect of the GATA4-null allele alone, which resulted in only a 4.3% rate of lung lobulation defects in *Frem1* ^eyes2/+^ mice. This suggests that *Frem1* and *Gata4* interact genetically in the development of lung lobulation defects and that *Gata4* expression can modulate the penetrance of this FREM1-related phenotype in mice.

Lungs from *Gata4* ^ki/ki^ embryos fail to form accessory lobes in culture due to an abnormality in branching morphogenesis [28]. In contrast, the lung lobe fusion defects seen in *Frem1* ^eyes2/+^;*Gata4* ^+/−^ and *Frem1* ^eyes2/eyes2^;*Gata4* ^+/−^ mice did not affect the accessory lobe and are similar to those seen in FRAS1-deficient mice, which have been attributed to defects in the basement membrane of the visceral pleura surrounding the developing lung [10]. At E14.5, GATA4 is expressed in the nuclei of mesothelial cells surrounding the developing lungs while *Frem1* is expressed in the mesenchymal cells that underlie the basement membrane of the visceral pleura. This makes it unlikely that GATA4 directly regulates the expression of *Frem1*. However, GATA4 may regulate the expression of *Fras1* and/or *Frem2* in a cell-autonomous fashion since these genes are expressed in mesothelial cells.

### *Slit3* as a Modifier of Renal Agenesis in FREM1-deficient Mice

Although *Slit3*-null mice have central diaphragmatic hernias–which are anatomically different than the retrosternal anterior hernias seen in *Frem1* ^crf11/eyes2^ and *Frem1* ^eyes2/eyes2^ mice–a similar pattern of decreased cell proliferation in the central tendon region of the diaphragm has been documented in both *Slit3-*null mice and *Frem1* ^eyes2/eyes2^ mice [12], [29], [30]. *Slit3*-null mice also have unilateral and bilateral renal agenesis that is similar to that seen in FREM1-deficient mice [29], [30]. This prompted us to look for evidence of a genetic interaction between *Frem1* and *Slit3*.

No increase in CDH prevalence was seen in *Frem1* ^eyes2/eyes2^ mice with the addition of a disrupted *Slit3* allele. In contrast, the prevalence of renal agenesis increased significantly from 2.7% in *Frem1* ^eyes2/eyes2^ mice to 13.6% in *Frem1* ^eyes2/eyes2^;*Slit3* ^+/−^ mice (p<0.02). This increase was more that could be attributed to the additive effect of the disrupted *Slit3* allele, since renal agenesis was not seen in *Frem1* ^eyes2/+^;*Slit3* ^+/−^ mice (0/126). This suggests that *Frem1* and *Slit3* interact genetically in the development of renal agenesis and that *Slit3* expression can modulate the penetrance of this FREM1-related phenotype in mice.

During normal development of the metanephric kidney, the ureteric bud extends out from the mesonephric duct and penetrates the metanephric mesenchyme. FRAS1, FREM1 and FREM2 are expressed around the ureteric bud at E11 and appear to undergo reciprocal stabilization as previously demonstrated in the skin [2], [59]. In FRAS1-deficient mice, lack of ureteric bud initiation from the mesonephric duct, or failure of the ureteric bud to penetrate the metanephric mesenchyme, causes the metanephric mesenchyme to undergo fulminant apoptosis, resulting in renal agenesis [59], [60]. Apoptosis of cells in the metanephric mesenchyme, leading to renal agenesis, is also seen in mice that lack the cytosolic adapter protein GRIP1, which plays a critical role in trafficking FRAS1 and FREM2 to the plasma membrane [2], [61]. It is possible that a similar mechanism underlies the development of renal agenesis in FREM1-deficient mice.

*In situ* hybridization studies performed at E12.5 demonstrate *Frem1* expression in the metanephric mesenchyme surrounding the growing uretic bud [1]. It is likely that the FREM1 protein detected around the ureteric bud during kidney development is secreted from these cells. Whole mount *in situ* hybridization studies of kidney explant cultures from E11.5 embryos do not detect *Slit3* transcripts in the invading ureteric tree or in the metanephric mesenchyme. However, *Slit3* transcripts were found at high levels in the intermediate mesoderm surrounding the metanephric mesenchyme after one day in culture (E12.5) [62]. Hence, *Frem1* and *Slit3* appear to be expressed in adjacent cell populations in the developing kidney. Further studies will be needed to determine the molecular mechanism by which decreased SLIT3 expression causes renal agenesis in *Slit3*-null mice and modulates the prevalence of renal agenesis caused by FREM1 deficiency.
